# A novel anti-HBV agent, *E*-CFCP, restores Hepatitis B virus (HBV)-induced senescence-associated cellular marker perturbation in human hepatocytes

**DOI:** 10.1016/j.virusres.2023.199094

**Published:** 2023-03-23

**Authors:** Yuki Takamatsu, Sanae Hayashi, Hiroki Kumamoto, Shuhei Imoto, Yasuhito Tanaka, Hiroaki Mitsuya, Nobuyo Higashi-Kuwata

**Affiliations:** a Department of Refractory Viral Diseases, National Center for Global Health and Medicine Research Institute, 1-21-1 Toyama, Shinjuku, Tokyo, 162-8655 Japan; b Department of Gastroenterology and Hepatology, Faculty of Life Sciences, Kumamoto University, 1-1-1 Honjo, Chuo, Kumamoto, 860-8556 Japan; c Department of Virology and Liver Unit, Nagoya City University Graduate School of Medical Sciences, 1 Kawasumi, Mizuho-cho, Mizuho, Nagoya, 467-8601 Japan; d Department of Pharmaceutical Sciences, Nihon Pharmaceutical University, 10281 Komuro, lna-machi, Kitaadachi-gun, Saitama, 362-0806 Japan; e Faculty of Pharmaceutical Sciences, Sojo University, 4-22-1 Ikeda, Nishi, Kumamoto 860-0082 Japan; f Experimental Retrovirology Section, HIV and AIDS Malignancy Branch, National Cancer Institute, National Institutes of Health, 10 Center Drive, Room 5A11, Bethesda, MD 20892-1868 USA; g Department of Clinical Sciences, Kumamoto University Hospital, 1-1-1 Honjo, Chuo, Kumamoto, 860-8556 Japan

**Keywords:** Hepatitis b virus, Nucleoside analogs, Cellular senescence, Human hepatocytes, p21^CIP1^

## Abstract

Cellular senescence is a cellular state with a broad spectrum of age-related physiological conditions that can be affected by various infectious diseases and treatments. Therapy of hepatitis B virus (HBV) infection with nucleos(t)ide analogs [NA(s)] is well established and benefits many HBV-infected patients, but requires long-term, perhaps lifelong, medication. In addition to the effects of HBV infection, the effects of NA administration on hepatocellular senescence are still unclear. This study investigated how HBV infection and NA treatment influence cellular senescence in human hepatocytes and humanized-liver chimeric mice chronically infected with live HBV. HBV infection upregulates or downregulates multiple cellular markers including senescence-associated β-galactosidase (SA-β-Gal) activity and cell cycle regulatory proteins (*e.g.*, p21^CIP1^) expression level in hepatocellular nuclei and humanized-mice liver. A novel highly potent anti-HBV NA, *E*-CFCP, *per se* did not have significant disturbance on markers evaluated. Besides, *E*-CFCP treatment restored HBV-infected cells to their physiological phenotypes that are comparable to the HBV-uninfected cells. The results reported here demonstrate that, regardless of the mechanism(s), chronic HBV infection perturbates multiple senescence-associated markers in human hepatocytes and humanized-mice liver, but *E*-CFCP can restore this phenomenon.

## Highlights

• HBV infection induces multiple cellular phenotypes in fresh human hepatocytes.
• A therapeutic candidate, *E*-CFCP restored the HBV-induced phenotypes.
• The NAs treatment alone except *E*-CFCP, induces changes in the senescence markers.

## 1 Introduction

Although the implementation of effective vaccination, hepatitis B virus (HBV) infection is still one of the greatest global public health problems which causes liver cirrhosis and hepatocellular carcinoma (HCC) (Alberts et al., 2022). Antiviral therapy should be given to most patients with life-threatening liver disease, compensated cirrhosis, or in the immune-active phase (Terrault et al., 2018; Kitayama et al., 2019). Oral anti-viral nucleos(t)ide analogs [NA(s)] have been shown to reduce HBV-related HCC incidence and improve prognosis (Lampertico P, Gastroenterology. 2019); however, the optimal duration of NAs is not well established (Papatheodoridis et al., 2016; Yoon EL, J Gastroenterol Hepatol. 2022) and long-term treatment is especially important for patients with cirrhosis (Chang et al., 2015). Although monotherapy with a single antiviral agent or interferon is unlikely to be sufficient for the eradication of HBV infection due to the drug resistance (Richman DD, Hepatology. 2000) or less hepatitis B surface antigen (HBsAg) seroclearance rate (Hsu et al., 2021), few combination therapies had shown to have additive activity against HBV or to prevent the emergence of drug resistance and none have been approved for routine use (Terrault et al., 2018). In this regard, even though multiple investigational therapies are in development to reduce the need for lifelong treatment (Dusheiko et al., 2023), evaluation of the long-term safety of anti-HBV therapeutics is still crucial.

Cellular senescence is a cell state implicated in various physiological processes and a broad spectrum of age-related diseases (Gorgoulis et al., 2019) which is also influenced by various infectious diseases and drug treatments (Kohli et al., 2021; Lee et al., 2021). Cellular senescence is characterized by a prolonged and usually irreversible cell cycle arrest with secretory characteristics, macromolecular damage and altered metabolism. In particular, the following markers are widely used for the evaluation of cellular senescence: ⅰ) Senescence-associated β-galactosidase (SA-β-gal) is one of the most common markers of senescence, which is prominent in senescent cells; ⅱ) cell cycle regulatory proteins (*e.g.*, cyclin-dependent kinase inhibitors p16^INK4a^, p21^CIP1^, and p27^KIP1^) act at the cell cycle checkpoint from G1 to S phase. Accumulation of these proteins results in persistent activation of Retinoblastoma (Rb) family proteins and consequent cell cycle arrest; ⅲ) senescence-associated secretory phenotype (SASP) is a phenomenon in which senescent cells secrete high levels of inflammatory cytokines and immunomodulatory factors through the DNA damage via the transcription factor p65^NF-κB^; ⅳ) Histone H2A.X belongs to the histone H2A family and reflects DNA damage and double-stranded DNA breaks. Phosphorylated histone H2A.X, γ-H2AX functions to recruit DNA repair. Cellular senescence also plays an important role in tumor suppression by its reduced cell proliferation (Herranz N, J Clin Invest. 2018; Chaib et al., 2022); however, persistent accumulation of senescent cells modulates the tissue microenvironment and may lead to age-related cancers (Igarashi et al., 2022), non-alcohol-related fatty liver disease (NAFLD) (Aravinthan et al., 2013; He et al., 2023), and viral HCCs (Giannakoulis et al., 2021). Patients with chronic HBV infection had epigenetic age acceleration (EAA) validated with DNA methylation (Horvath's clock [Horvath S, Genome Biol. 2013]), and one year NA treatment reduced such EAA (Gindin et al., 2021). Individuals with hepatitis C virus (HCV) also had significant EAA and HCV elimination with direct-acting antivirals (DAAs) therapy resulted in decreased EAA likewise (Oltmanns et al., 2023). Studies have suggested that cellular senescence may enhance antiviral immunity by increasing antiviral cytokines, while other evidence shows that senescence may enhance viral replication by downregulating antiviral signaling (Kelley et al., 2020). Thus, hepatic senescence may have both beneficial (cancer-protective due to reduced cell proliferation) and detrimental (cancer-promoting due to modulation of the tissular microenvironment) effects (Huda et al., 2019; Karakousis et al., 2020). In this context, pharmacological approaches to eliminate senescent cells have been under investigation (Zhang et al., 2022); however, the effect of antiviral therapy on cell aging is complex and the effects of long-term administration of the NAs and HBV infection itself on cellular senescence have not yet clearly evaluated.

We previously reported a long-acting anti-HBV therapeutic candidate, *E*-CFCP, a potent NA active against drug-resistant HBV in HBV-infected human-liver-chimeric mice (PXB-mice) with minimal toxicity (Higashi-Kuwata et al., 2021). *E*-CFCP is one of the congeners of islatravir (ISL/EFdA), which has a unique 4´-ethynyl moiety and exerts highly potent anti-human immunodeficiency virus (HIV)-1 activity both *in vitro* (Nakata et al., 2007) and *in vivo* (Hattori et al., 2009). This unique 4´-ethynyl moiety plays a crucial role in a potent activity of EFdA against multiple nucleoside reverse transcriptase inhibitor (NRTI)-resistant HIV-1s (Takamatsu et al., 2018). In this regard, we have previously shown that the 4´-modification with a cyano moiety results in a potent inhibitor of both HBV and HIV-1 (Takamatsu et al., 2015; Higashi-Kuwata et al., 2019). In particular, *E*-CFCP has been developed for potential clinical application with high potency against drug-resistant viruses and persistence for 1–3 weeks after cessation of treatment (Higashi-Kuwata et al., 2021). Although long-term data indicate that currently available NA treatments suppress covalently closed circular DNA (cccDNA) (Werle-Lapostolle et al., 2004; Lai et al., 2017), none have eliminated cccDNA and therefore a life-long treatment is necessary. Thus, it is highly desirable to develop potent therapeutics which could fulfill 100% inhibition of HBV replication and prevention of recycling cccDNA with ideally no side effects (Durantel et al., 2021). Here, to further investigate the safety of NA treatment, we aim to evaluate the effect of HBV infection and NA treatment, including *E*-CFCP, in human hepatocytes on senescence-associated cellular phenotypes *in vitro* and *in vivo*.

## 2 Materials and methods

### 2.1 Cells, viruses, and antiviral agents

Fresh human hepatocytes (PXB-cells) derived from humanized-liver chimeric mice (PXB-mice) were purchased from PhoenixBio Co., Ltd. (Hiroshima, Japan). PXB-mice are severe combined immunodeficiency (SCID) mice that are transgenic for the urokinase-type plasminogen activator (uPA) gene with more than 70% of their liver replaced with human hepatocytes (Tateno et al., 2004; Yamasaki et al., 2010). More than 90% of the PXB-cells isolated from PXB-mice consisted of human hepatocytes. The sources for infectious HBV (mice sera, Lot# 210,322) were obtained from PXB-mice previously infected with genotype C_AT HBV and purchased from PhoenixBio. (1*S*,3*S*,5*S,E*)−3-(2-amino-6-oxo-1,6-dihydro-9*H*-purin-9-yl)−2-(fluoromethylene)−5‑hydroxy-1-(hydroxymethyl)cyclopentane-1-carbonitrile (*E*-CFCP) was synthesized by Kumamoto et al. (Higashi-Kuwata et al., 2021) with 99.2% purity determined by using high-performance liquid chromatography (HPLC). The detailed synthesis methods for *E*-CFCP will be reported elsewhere by Kumamoto and others. Entecavir (ETV) was purchased from Tokyo Chemical Industry Co., Ltd. (Tokyo, Japan). Tenofovir disoproxil fumarate (TDF) was purchased from FUJIFILM Wako Pure Chemical Co., Ltd. (Osaka, Japan).

### 2.2 *In vitro* viral infection assay

**Fig. 1:**
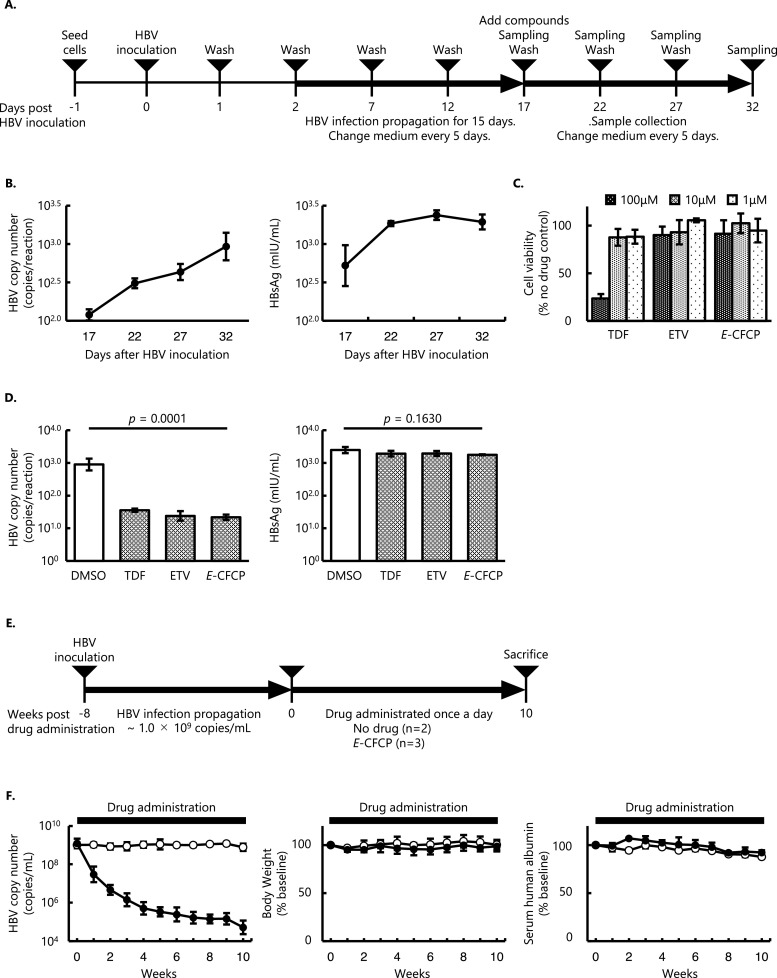
**Experimental setup for *in vitro* and *in vivo* assays. (A)** Schema of the *in vitro* HBV infection model. **(B)** The kinetics of HBV-DNA copy numbers and the amounts of HBsAg in the supernatants without NA treatment. Values shown are geometric means ±  standard deviation (SD) (*n* = 3) **(C)** Cytotoxicity assay of NAs *in vitro* using MTT assay. The cell viability in each experimental group was expressed as a percentage relative to the absorbance level of no drug control as 100% (*n* = 3) **(D)** HBV-DNA copy numbers and the amounts of HBsAg in the supernatants at the end of the study. Geometric means ± SD are shown (*n* = 3). **(E)** Schema of the *in vivo* HBV infection model. **(F)** Changes in HBV copy numbers, body weights, and serum human albumin levels from the baseline (immediately before the drug administration). *E*-CFCP continuously blocked the replication of the virus until the end of the study observation period, without any significant systemic condition changes. Thick bars denote the administration period (10 weeks) with 0.2 mg/kg/day. Open and closed circles indicate no drug control and *E*-CFCP-treated mice, respectively.

PXB-cells were seeded on type I collagen-coated 96-, 48-, or 12-well plates at a density of 2.1 × 10^5^ cells/cm^2^. On the following day, PXB-cells were infected with HBV at a multiplicity of infection (MOI) of 5 in the presence of 4% polyethylene glycol (PEG) 8000 (Promega, Madison, WI) in 50 to 400 μL of hepatocyte growth medium (PhoenixBio). On 1 and 2 days after infection, the PXB-cells were washed with the fresh medium, and 0.2 to 2 mL of the fresh hepatocyte growth medium was added. The culture medium was collected and replenished every 5 days. The PXB-cells were cultured under humified conditions containing 5% CO_2_ at 37 °C. After a total of 17 days of HBV propagation among the cells, an anti-HBV compound (TDF, ETV, or *E*-CFCP) or vehicle (0.05% dimethyl sulfoxide [DMSO]) was added, and the compound exposure was continued throughout the study. The schema of the *in vitro* HBV infectious assay is presented in Fig. 1A.

### 2.3 *In vivo* viral infection assay

Male uPA/SCID mice between 12 and 18 weeks of age with humanized liver (PXB-mice) were generated as previously described by PhoenixBio, Co. Ltd. (Japan) (Tateno et al., 2004; Yamasaki et al., 2010). Briefly, frozen human hepatocytes (donor BD195, Corning Incorporated, Tewksbury, MA) were transplanted into 2- to 4-week-old uPA/SCID mice by splenic injection. The mice with an estimated liver replacement index of human hepatocytes greater than 70% were selected for studies. The mice were infected with genotype C_AT HBV at 1.0 × 10^6^ copies/body at 12 weeks old. Approximately 8 weeks after HBV infection, when the serum HBV copy number reached approximately 1.0 × 10^9^ copies/mL, the mice were randomly selected into study groups. General health observations including body weights and serum human albumin levels were monitored weekly. All animal protocols were performed following the Guide for the Care and Use of Laboratory Animals and approved by the Animal Welfare Committee of Phoenix Bio Co., Ltd. All mice were housed individually and maintained following the Animal Ethics Committee of PhoenixBio (resolution # 2507). The schema of the *in vitro* HBV infectious assay is presented in Fig. 1E.

### 2.4 Quantification of HBV DNA copy numbers and the hepatitis B surface antigen (HBsAg)

The DNA samples of the cell culture supernatants and the harvested cells from *in vitro* experiments were prepared using QIAamp DNA Blood Mini Kit (QIAGEN, Hilden, Germany) according to the manufacturer's instructions and were re-suspended in 100 µL Tris-ethylenediaminetetraacetic acid (EDTA) buffer. The amount of HBV DNA was determined with the quantitative polymerase chain reaction (qPCR) assay using genesig standard Kit for HBV core protein region (Primerdesign, Camberley, UK) as previously described (Higashi-Kuwata et al., 2021). The DNA samples from *in vivo* assays were prepared from 5 μL of murine serum using SMITEST EX-*R* + *D* KIT (Medical and Biological Laboratories, Tokyo, Japan) according to the manufacturer's instructions. The amount of HBsAg was measured using a fully automated chemiluminescent enzyme immunoassay (CLEIA) system (Lumipulse F; Fujirebio, Tokyo, Japan), as previously described (Higashi-Kuwata et al., 2021). The detection limit of the HBsAg in the apparatus is 0.005 IU/ml.

### 2.5 Detection of senescence-associated β-galactosidase (SA-β-Gal) activity

The cell staining was performed using Senescence β-Galactosidase Staining kit (Cell Signaling Technology, Danvers, MA) according to the manufacturer's instructions. Briefly, the PXB-cells were fixed with 1× fixation solution at room temperature for 15 min. The fixed cells were rinsed two times with 1× phosphate-buffered saline (PBS) and stained with β-Galactosidase Staining Solution at 37 °C (without CO_2_) overnight. The activity of SA-β-Gal from three independent experiments was quantitated using 96-Well Cellular Senescence Assay Kit (Cell Biolabs, San Diego, CA) according to the manufacturer's instructions. The resulting fluorescence was normalized to the concentration of the protein as measured by using Pierce BCA Protein Assay Kit (Thermo Fisher Scientific, MA, USA).

### 2.6 Traditional western blot analysis

The nuclear proteins of PXB-cells were extracted using Minute Cytoplasmic and Nuclear Extraction Kit for Cells (Invent Biotechnologes, Plymouth, MN), while the whole liver proteins of PXB-mouse were extracted with Minute Total Protein Extraction Kit for Animal Cultured Cells and Tissues (Invent Biotechnologes) according to the manufacture instructions. Protein concentration was determined with Pierce BCA Protein Assay Kit (Thermo Fisher Scientific). Samples (2 µg/well) were run at in 8–16% Mini-PROTEAN TGX gels (Bio-Rad, Hercules, CA, USA) using a Mini-PROTEAN Tetra Cells (Bio-Rad) and Trans-Blot Turbo Transfer System (Bio-Rad) was used for transfer onto 0.2 µm PVDF membranes (Bio-Rad). Membranes were blocked with 5% bovine serum albumin (BSA) in TRIS-buffered saline, 0.1% tween 20 (TBST), and incubated with diluted primary antibody at 4 °C overnight. After twice washing, the membranes were incubated with HRP-conjugated secondary antibody, and the detected signals using SignalFire ECL Reagent (Cell Signaling, Danvers, MA, USA) were captured using iBright CL1500 Imaging System (Thermo Fisher Scientific).

### 2.7 Capillary-based western blot analysis

The amounts of Hepatitis B Virus-encoded X protein (HBx), p16^INK4a^, p21^CIP1^, p27^KIP1^, p38^MAPK^, and Lamin B1 were quantified using the capillary electrophoresis Simple Western Jess apparatus (Protein Simple, San Jose, CA) according to the manufacturer's instructions as previously described (Takamatsu et al., 2022). In brief, approximately 0.5 mg/mL of nuclear proteins of PXB-cells and 5 mg/mL of whole liver protein of PXB-mouse were analyzed. Proteins were covalently fixed with UV irradiation to a 12–230 kDa Jess & Wes Separation Module (Protein Simple) capillaries. The immobilized proteins were then exposed to each 50-fold diluted primary antibody; Anti-Hepatitis B Virus X antigen antibody [X36C] #ab2741 (abcam, Cambridge, UK), p16 INK4A (D7C1M) Rabbit mAb #80,772, p21 Waf1/Cip1 (12D1) Rabbit mAb #2947, p27 Kip1 (D69C12) XP Rabbit mAb #3686, p38 MAPK Antibody #9212, p53 (7F5) Rabbit mAb #2527, and Lamin B1 (D4Q4Z) Rabbit mAb #12,586 (Cell Signaling Technology, Danvers, MA). Subsequently, the antibodies bound to each target protein were probed with horseradish peroxidase (HRP)-conjugated anti-rabbit secondary antibody (Protein Simple). The presence of each protein in the capillary was detected by iridescent light produced by the luminol reagent being mediated by HRP. The obtained chemiluminescence was normalized with the total protein concentration using Protein Normalization Detection Module (Protein Simple). The signal intensity was quantified and plotted as a peak graph in the analysis software (Compass for SW ver. 6.2.0, Protein Simple). The detection limit of HBx and p16^INK4a^ was determined using serially diluted reference proteins; Recombinant Hepatitis B virus Protein X #ab203540 (abcam) and p16INK4A (CDKN2A)(NM_000077) Human Recombinant Protein #TP320937 (ORIGENE, Rockvill, MD) were used as a positive control. The analysis was conducted using samples from two independent experiments.

### 2.8 Immunocytochemistry analysis

PXB-cells in 96-well microculture plates were fixed in 4% paraformaldehyde (PAF) for 15 min, permeabilized with 0.5% Triton X-100 in PBS for 15 min, and blocked with 3% bovine serum albumin in PBS for 60 min. All the procedures were performed at room temperature. Cells were then incubated with primary antibodies: anti-p21^CIP1^ (12D1)(1:800, rabbit monoclonal, #2947, Cell Signaling Technology, MA) and anti-HBsAg (1:50, goat polyclonal, bs-1557 G, Bioss Antibodies, Woburn, MA), at 4 °C overnight. The stained cells were washed with PBS (300 µl/well) three times for 5 min each time, and the cells were incubated with secondary antibodies: Donkey anti-Rabbit IgG (*H* + *L*) Alexa 488 (Jackson ImmunoResearch Laboratories, West Grove, PA) and Donkey anti-Goat IgG (*H* + *L*) Alexa 594 (Jackson ImmunoResearch Laboratories), for double staining for 2 h. After washing the cells with PBS (300 µl/well) three times for 5 min each time, 4′,6-diamidino-2-phenylindole (DAPI) solution (Thermo Fisher Scientific, Waltham, MA)-PBS (50 µl/well) was added to stain nuclei. Fluorescent signals were acquired with Cytation 5 cell imaging multimode reader (BioTek Instruments, Winooski, VT). The number of positively stained cells in p21^CIP1^ staining was counted in three randomly selected areas in 100-fold magnification using Gen5 software (BioTek Instruments). The anti-p21^CIP1^ rabbit monoclonal antibody, which detects only human and monkey p21^CIP1^, was validated using immunohistostaining of human and murine liver cells in PXB-mice, and the data obtained were confirmed to be free of non-specific detection. Similarly, the anti-HBsAg goat polyclonal antibody was validated using immunocytostaining of HBV-infected and -uninfected PXB-cells, and the data obtained were confirmed to be free of non­specific detection.

### 2.9 Immunohistochemistry analysis

Paraffin sections with a thickness of 3 µm were processed for immunohistochemistry. For labeling with primary antibodies, the sections were deparaffinized and treated for 30 min with 0.3% H_2_O_2_ in methanol. All the sections were heated in an autoclave for 20 min in citrate buffer (pH 6.0), then incubated with the primary antibodies overnight. The primary antibody and conditions used were as follows; anti-p21^Waf1/Cip1^ (12D1)(1:50 rabbit monoclonal, #2947, Cell Signaling Technology). Sections were stained with biotinylated anti-rabbit IgG (Dako, CA, USA) and visualized using H_2_O_2_-containing diaminobenzidine buffer. The anti-p21^CIP1^ rabbit monoclonal antibody was validated as described above, in ***2.8 Immunocytochemistry analysis*** section.

### 2.10 Quantification of human interleukin-6 (IL-6), IL-8, and reactive oxygen species (ROS) activity

The amounts of SASP (IL-6 and IL-8) in cell culture supernatant from three independent experiments were determined using Human IL-6 Quantikine ELISA Kit and Human IL-8/CXCL8 Quantikine ELISA Kit (R&D Systems, Minneapolis, MN), respectively. The activity of reactive oxygen species (ROS) was determined using Cell Meter Fluorimetric Intracellular Total ROS Activity Assay Kit*Green Fluorescence* (ATT Bioquest, Sunnyvale, CA). In brief, the day after the PXB-cells seeding in 96-well type I collagen-coated plates, 100 μL of Amplite ROS Green working solution was added to each well containing 100 μL cell culture medium and incubated for 1 hour. After adding a compound (TDF, ETV, or *E*-CFCP; final concentration 10 μM), vehicle (DMSO; final concentration 0.05%), or positive control (H_2_O_2_; final concentration 1 mM), the cells were cultured for 7 days in humified conditions containing 5% CO2 at 37 °C. The fluorescence was measured by excitation/emission 490 nm/525 nm using Cytation 5 cell imaging multimode reader (BioTek Instruments).

### 2.11 Statistical analysis

All the summary statistics were calculated using Microsoft Excel and all the statistical analyses were performed using R version 4.2.2 (R Core Team 2022). Two-sided unpaired Student's *t*-test was used to compare the differences between groups and *p*-values were calculated. The statistical significance threshold was *p*<0.05.

## 3 Results

### 3.1 Successful HBV replication in chronic infection model *in vitro* and *in vivo*

Fresh human hepatocytes (PXB-cells) derived from humanized chimeric mice (PXB-mice) were exposed to genotype C wild-type HBV (Ishida et al., 2015) (Fig. 1A). Increased HBV-DNA copy number and HBsAg levels in the culture supernatants of no drug control indicated the successful propagation of HBV infection among the human hepatocytes during the experimental period (Fig. 1B). After determining the cytotoxic concentration of each compound in PXB-cells (Fig. 1C), three anti-HBV NAs including the long-acting therapeutic candidate, *E*-CFCP (Higashi-Kuwata et al., 2021), under a nontoxic concentration (10 µM) were administrated to the cells. Each compound significantly blocked the replication of HBV after 15 days of treatment, while the HBsAg levels were not suppressed by NAs (Fig. 1D).

Viral replication has also been successfully demonstrated in humanized-liver mice (PXB-mice) with genotype C wild-type HBV infection *in vivo* (Ishida et al., 2015; Takamatsu et al., 2015; Higashi-Kuwata et al., 2019, J Hepatol. 2021). After 8 weeks of HBV propagation, 0.2 mg/kg/day of *E*-CFCP was administrated intragastrically once a day for 10 weeks (Fig. 1E). *E*-CFCP significantly and continuously suppressed the HBV viremia until the end of the observation period (closed circle) (Fig. 1F, left panel). In contrast, the virus copy number in mice sera without any treatment was consistent throughout the 10-week experimental period (open circle) (Fig. 1F, left panel). Continuous HBV infection or *E*-CFCP administration did not worsen the systemic conditions of mice during the observation period and the behavior, body weights, and serum human albumin levels of the enrolled PXB-mice were consistent (Fig. 1F, middle and right panels).

### 3.2 HBV infection perturbates multiple senescence-associated markers in human hepatocytes and these markers can be restored by NA treatment *in vitro*

**Fig. 2:**
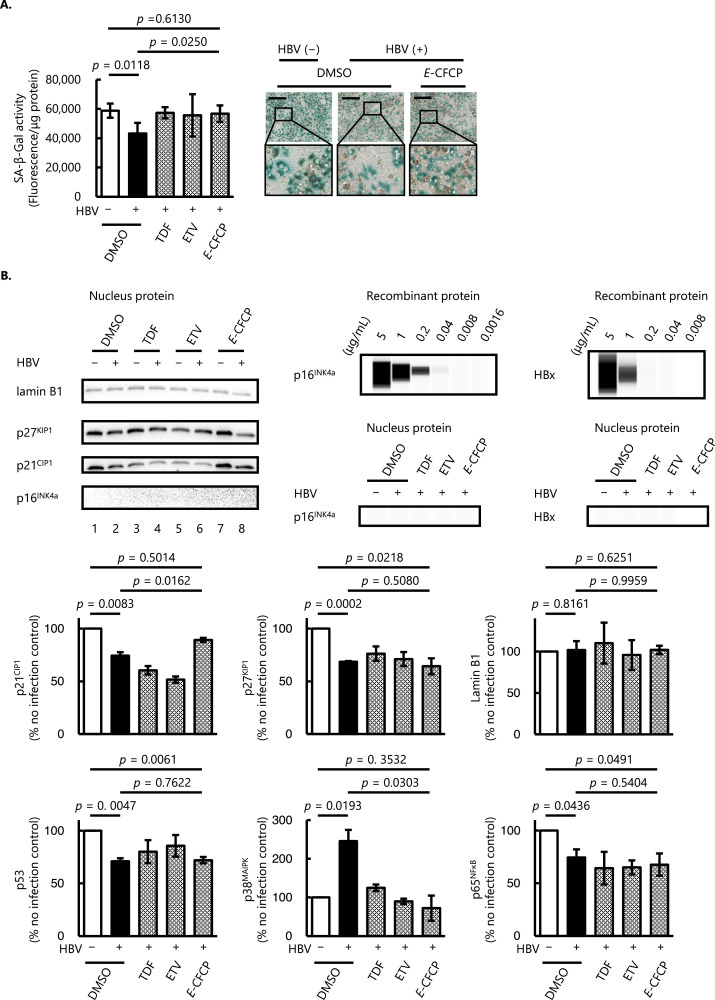

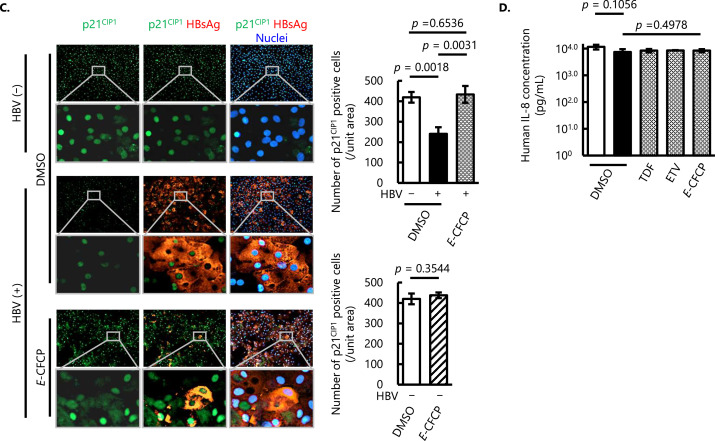
**Senescence-associated markers in HBV-infected human hepatocytes *in vitro*. (A)** Senescence-associated β-galactosidase (SA-β-Gal) activity in human hepatocytes. Columns and vertical lines denote arithmetic means and SD values, respectively (*n* = 3). Representative bright field images of SA-β-Gal staining are shown in the right panel. SA-β-Gal positive signals are observed in blue. Brown conglomerates in the images represent lipofuscins (one of the aging or "wear-and-tear" pigments) observed regardless of the present experiments. Scale bar, 200 μm. The framed areas show the magnified images. **(B)** Western blot (WB) analysis of nuclear proteins related to cell cycles (p16^INK4a^, p21^CIP1^, p27^KIP1^) and nuclear membrane (Lamin B1). The analysis was conducted using samples from two independent experiments and the representative blot images are shown (upper left panel). Since we failed to detect p16^INK4a^ and HBx proteins with the traditional WB analysis, we performed a high-sensitivity capillary-based WB analysis. Concentration dependent signal was detected both in recombinant p16^INK4a^ and HBx proteins analysis with a detection limit of 0.04 and 0.2 μg/mL, respectively; however, no signal was detected in nuclei samples even in the high sensitivity analysis (upper right panel). In the lower panel, quantification of cell cycle regulator proteins and transcription factors levels in PXB-cells’ nuclei are shown (average ± S.D. from two independent assays). **(C)** Immunocytochemical (ICC) analysis of HBV-uninfected hepatocytes with no drug treatment (top panels), HBV-infected hepatocytes with no drug treatment (middle panels), and HBV-infected hepatocytes with *E*-CFCP treatment (bottom panels). Alexa 488-labeled p21^CIP1^, Alexa 594-labeled HBsAg, and nuclei are visualized by green, red, and blue fluorescence, respectively. Representative morphological images from three independently conducted experiments are shown. The framed areas show magnified images. HBV infection caused a significant decrease in p21^CIP1^-positive cell counts, but *E*-CFCP administration fully restored them to the uninfected physiological level (right panel). **(D)** Amounts of one of the senescence-associated secretory phenotypes (SASP), IL-8, in the culture supernatant. Values shown are arithmetic means ± SD (*n* = 3). The groups of HBV-uninfected hepatocytes with no drug treatment, HBV-infected untreated hepatocytes, HBV-infected NA-treated hepatocytes, and HBV-uninfected NA-treated hepatocytes are shown in open, closed, shaded, and diagonal stripes columns, respectively.

We first evaluated the effect of HBV infection and NA treatment in human hepatocytes *in vitro* using multiple cellular senescence markers such as SA-β-Gal activity, cell cycle regulator proteins, transcription factors, and SASPs (Gorgoulis et al., 2019). In HBV-infected human hepatocytes, SA-β-Gal activity was significantly lower than in the cells without HBV infection (*p* = 0.0118), while this reduced SA-β-Gal activity was restored to compatible levels with HBV-uninfected cells after 15 days of *E*-CFCP treatment (Fig. 2A).

To investigate the transcriptional indications, we extracted nuclear proteins from the PXB-cells and conducted Western blotting (WB) analysis on the cell cycle regulator proteins including cyclin-dependent kinase inhibitor CDKN1A (p21^CIP1^), CDKN2A (p16^INK4a^), and CDKN1B (p27^KIP1^) (Fig. 2B). Of note, the expression level of p21^CIP1^ was decreased in HBV-infected human hepatocytes nuclei (Lane 2) compared with that in HBV-uninfected cells (Lane 1). The decreased p21^CIP1^ expression in response to HBV infection was not reversed by either of the two currently available HBV therapeutic NAs, TDF or ETV (Lane 4 and 6, respectively), while administration of *E*-CFCP (Lane 8) restored the expression level to that of virus-free no-drug (0.05% DMSO)-treated control cells (Lane 1) (Fig. 2B). On the other hand, there was no reversal of the decreased p27^KIP1^ expression following HBV infection by all the NAs evaluated (Lane 4,6,8). There was no significant difference in the expression levels of Lamin B1. Since we did not detect p16^INK4a^ signals with conventional WB analysis, we conducted capillary-based WB analysis, which possesses higher sensitivity in protein detection as previously described (Takamatsu et al., 2022). We confirmed the concentration-dependent chemiluminescence signal with diluted recombinant p16^INK4a^ protein (detection limit 0.04 μg/mL); however, no p16^INK4a^ signal was detected in nucleus protein samples even in such high-sensitivity capillary-based WB analysis, suggesting the concentration of p16^INK4a^ in the sample was below 0.04 μg/mL (Fig. 2B). We also performed the capillary-based WB analysis to quantify such cell cycle regulator proteins and transcription factors (Fig. 2B). The expression level of transcription factor p53 and nuclear factor-kappa B (p65^NFκB^) was also significantly decreased in HBV-infected PXB-cells nuclei and NA treatment did not restore such reduction as p27^KIP1^. In contrast, another transcription factor p38^MAPK^ was significantly upregulated in HBV-infected cells. This drastic increase was significantly suppressed by NA treatment (Fig. 2B). No HBx protein signal was detected in the nucleus protein, while concentration dependent chemiluminescence signal was observed with recombinant HBx protein (detection limit 0.2 μg/mL) (Fig. 2B).

The decreased p21^CIP1^ expression in HBV-infected human hepatocytes was also confirmed with immunocytochemistry (ICC). The number of p21^CIP1^-positive nuclei (labeled with green fluorescence, Alexa 488) was significantly lower in the HBV-infected cells than that in HBV-uninfected cells (*p* = 0.0018) (Fig. 2C). Note that HBV infection was confirmed with the presence of red fluorescence (Alexa 594)-labeled HBsAg in the cytoplasm (Fig. 2C). The number of p21^CIP1^-positive nuclei was significantly greater in *E*-CFCP-treated HBV-infected cells than that in the vehicle (0.05% DMSO)-treated HBV-infected cells (*p* = 0.0031), which was compatible with that of HBV-uninfected physiological hepatocytes (*p* = 0.6536) (Fig. 2C). Meanwhile, there was no significant difference in p21^CIP1^-positive cell numbers between the control and *E*-CFCP-treated HBV-uninfected cells (*p* = 0.3544) (Fig. 2C).

We also assessed the secretory phenotype of the human hepatocytes *in vitro* by evaluating the amounts of interleukin-6 (IL-6) and IL-8, which are controlled by the activation of transcription factors such as p65^NFκB^ and p38^MAPK^. IL-8 levels did not change in the current *in vitro* assay system, regardless of HBV infection or NA treatment (Fig. 2D). The IL-6 level was under detection limit in our assay (< 0.7 pg/mL).

### 3.3 HBV infection upregulated p21^CIP1^ in humanized-mice liver but *E*-CFCP restored the elevation

**Fig. 3:**
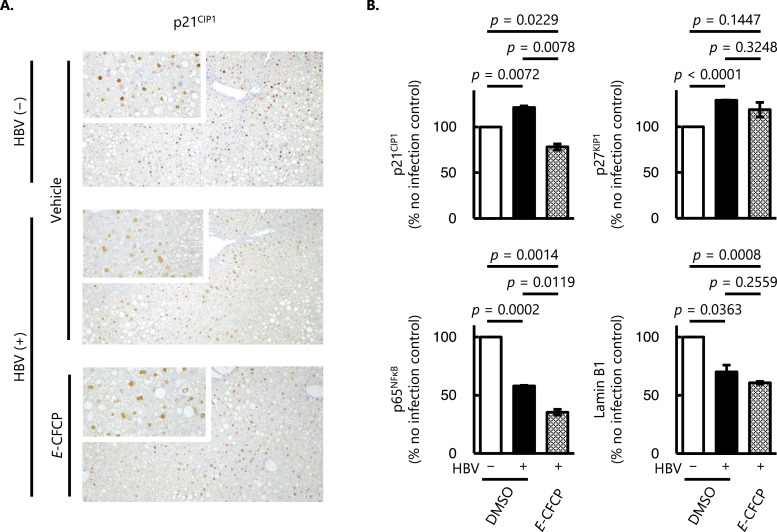
**Senescence-associated markers in HBV-infected liver *in vivo*. (A)** Immunohistochemical (IHC) analysis of liver slices from HBV-uninfected PXB-mice with no drug treatment (top panel), HBV-infected PXB-mice with no drug treatment (middle panel), and HBV-infected, *E*-CFCP-treated PXB-mice (bottom panel) was conducted. Representative morphological images from two independently conducted experiments are shown. Brown signals indicate p21^CIP1^ positive cells. The frames show magnified images. **(B)** Quantification of senescence-associated cell cycle regulatory proteins (p21^CIP1^ and p27^KIP1^), transcription factor (p65^NFκB^), and nuclear membrane (Lamin B1) using capillary-based WB analysis. The analysis was conducted using samples from two independent experiments and arithmetic means ± SD are shown. Contrary to the *in vitro* hepatocyte nuclei samples and IHC analysis, expression levels of p21^CIP1^ and p27^KIP1^ in whole liver samples were significantly higher in the HBV-infected un-treated mice. The groups of HBV-uninfected mice with no drug treatment, HBV-infected untreated mice, and HBV-infected NA-treated mice are shown in open, closed, and shaded columns, respectively.

To further validate the effect of HBV infection and anti-viral treatment with *E*-CFCP *in vivo*, we employed humanized-liver chimeric mice (PXB-mice) and evaluated the histopathological analysis of liver samples. The immunohistochemical (IHC) analysis showed fewer p21^CIP1^-positive nuclei and signal levels in HBV-infected human hepatocytes (Fig. 3A middle panel). In contrast, greater numbers or signals of p21^CIP1^-positive nuclei were observed in the liver of age-matched HBV-uninfected PXB-mice (Fig. 3A upper panel) and *E*-CFCP-treated mice (Fig. 3A bottom panel).

Contrary to the *in vitro* hepatocyte nuclei samples and IHC analysis, expression levels of p21^CIP1^ and p27^KIP1^ in whole liver samples were significantly higher in the HBV-infected un-treated mice compared to HBV-uninfected animals (Fig. 3B). In HBV-infected mice treated with *E*-CFCP, the level of p21^CIP1^ expression in the liver was significantly reduced than HBV-infected non-treated mice, while the level of p27^KIP1^ slightly, but not statistically significantly, reduced by the drug treatment (Fig. 3B). Consistent with hepatocyte nucleus WB analysis, HBV infection significantly downregulated p65^NFκB^ expression and *E*-CFCP treatment further suppressed the level. The expression and the response of Lamin B1 were also along the same line (Fig. 3B).

### 3.4 *E*-CFCP had no significant disturbance on senescence-associated markers in human hepatocytes

**Fig. 4:**
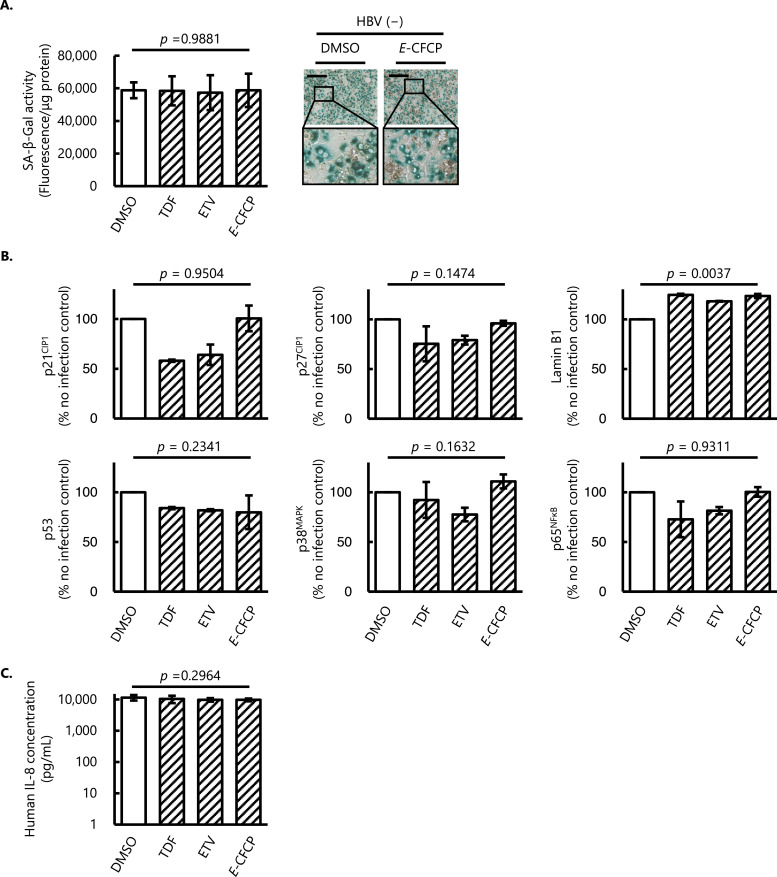
**Senescence-associated markers in HBV un-infected NAs-treated human hepatocytes *in vitro*. (A)** SA-β-Gal activity in human hepatocytes without HBV infection. Columns and vertical lines denote arithmetic means and SD values, respectively (*n* = 3). Representative bright field images of SA-β-Gal staining are shown in the right panel. Note that all the NAs tested had no significant effect on SA-β-Gal activity compared to vehicle (DMSO)-treated cells. Scale bar, 200 μm. The framed areas show the magnified images. **(B)** Capillary-based WB analysis of nuclear proteins related to cell cycles (p21^CIP1^ and p27^KIP1^), transcription factor (p53, p38^MAPK^, and p65^NFκB^), and nuclear membrane (Lamin B1). The analysis was conducted using samples from two independent experiments and the average ± SD are shown. *E*-CFCP had no significant disturbance on senescence-associated markers except for the elevation of Lamin B1. **(C)** Amounts of IL-8 in the culture supernatant. Values shown are arithmetic means ± SD (*n* = 3). The groups of HBV-uninfected hepatocytes with no drug treatment and HBV-uninfected NA-treated hepatocytes are shown in open and diagonal stripes columns, respectively.

We finally asked whether such phenotypes were directly introduced with NAs without HBV infection *in vitro*. NAs including *E*-CFCP alone did not affect the SA-β-Gal activity unlike the HBV-infected hepatocytes described above (Fig. 4A). Capillary-based WB analysis of human hepatocytes nucleus protein demonstrated significantly lower expression levels of p21^CIP1^ in TDF- and ETV-treated cells compared with those in the vehicle (0.05% DMSO)-treated cells. Meanwhile, the expression levels of all the cell cycle regulator proteins and transcription factors tested in *E*-CFCP-treated cells were compatible with those in vehicle-treated cells (Fig. 4B). Nucleus membrane protein Lamin B1 was only the one which increased in all the cells treated with NAs compared to that in the vehicle (0.05% DMSO)-treated cells (Fig. 4B). In terms of SASP, IL-8 levels did not change in NAs-treated human hepatocytes without HBV infection (Fig. 4C). We confirmed that the NAs examined did not enhance the reactive oxygen species (ROS) activity during the exposure (data not shown). These data, taken together, suggest that even under the physiological nontoxic concentration, conventional anti-HBV therapeutic NAs, TDF or ETV, downregulate the expression levels of p21^CIP1^ irrespective of whether the HBV infection (Fig. 2B, Fig. 4B), while such an effect was minimal or absent with *E*-CFCP.

## 4 Discussion

Studies using clinical liver biopsy samples have indicated the relationship between HBV infection and cell cycle dysregulation during disease progression (Kim et al., 2009; Tachtatzis et al., 2015). SA-β-Gal activity reflects the cytoplasmic lysosomal mass due to either the accumulation of old lysosomes or increased lysosomal biogenesis (Hernandez-Segura et al., 2018). Regarding HBV-related liver cirrhosis and HCC, SA-β-Gal activity was decreased in large cell liver change, which may represent a precursor lesion to hepatocellular carcinoma, and was absent in HCC (Kim et al., 2009; Ikeda et al., 2009). In the current study, we found that SA-β-Gal activity decreased in human hepatocytes chronically infected with HBV (for 32 days) *in vitro*, while NA treatment (for 15 days) restored the activity to levels comparable with non-HBV-infected hepatocytes (Fig. 2A). This result suggests that our *in vitro* HBV infection model recapitulates the tissue changes caused by chronic HBV infection and HBV-induced cellular state changes may reversibly return to physiological states by antiviral drug therapy.

To further investigate other senescence-associated cellular phenotypes in detail to understand this phenomenon, we extracted the nuclear proteins and conducted WB analysis of the cell cycle regulator proteins and transcription factors. Previous studies have indicated that p21^CIP1^ is upregulated in HBV-infected hepatocytes and mediates senescence and inflammation (Qiao et al., 2001; Chin et al., 2010; Torresi et al., 2019). In the current study, we found a significant increase of p21^CIP1^ levels in liver samples from HBV-infected humanized liver mice, and *E*-CFCP treatment significantly suppressed the p21^CIP1^ levels compared with HBV-uninfected animals (Fig. 3B). On the other hand, the analysis of nuclear proteins from *in vitro* human hepatocytes revealed that p21^CIP1^ levels were significantly decreased in HBV-infected hepatocytes, and only *E*-CFCP restored this reduction to levels like those of HBV-uninfected physiological cells (Fig. 2B, 2C). A possible explanation for this apparent contradiction is that the expression and location of cell cycle regulatory proteins may be different in the cytoplasm and the nucleus depending on the cell and viral infectious status. In fact, Yano et al. reported the elevated cytoplasmic p21^CIP1^ in HBx-overexpressing cells. However, interferon beta (IFN-β), an anti-hepatocarcinogenic agent treatment showed a shift of cytoplasmic p21^CIP1^ to the nucleus (Yano et al., 2013). Since the intracellular distribution of p21^CIP1^ appears to have distinct and opposing functions: to promote carcinogenesis when localized in the cytoplasm and to inhibit carcinogenesis when localized in the nucleus (Zhou et al., 2001), sorting and comparing nuclear and cytoplasmic protein levels has been suggested.

The mechanisms and activation of p21^CIP1^ in HBV infection are complex. Several factors have been proposed to affect its expression: ⅰ) p38^MAPK^/extracellular signal-regulated kinase (ERK) pathways (Chin et al., 2007), ⅱ) DNA damage (Zhao et al., 2008), or ⅲ) protein kinase B (PKB/Akt) (Chin et al., 2008). Of these factors, ⅳ) multifunctional HBV genome encoded HBx protein (Hayashi et al., 2021) has been reported to induce hepatocarcinogenesis by increasing cytoplasmic p21^CIP1^ and inhibiting cell cycle progression (Qiao et al., 2001; Yano et al., 2013). In contrast, another study has shown that HBx represses p21^CIP1^ expression (Ahn et al., 2001). Noteworthy, the effects of HBx on cell proliferation and carcinogenesis have largely varied depending on the cell line and assays used in the study (Slagle et al., 2015). For instance, using primary human hepatocytes infected with HBx-encoding adenovirus, Gearhart et al. demonstrated that HBx protein increases the levels of p21^CIP1^ and p27^KIP1^, while the levels of p16^INK4a^ decreased in hepatocytes lysates (Gearhart TL, Virus Res. 2011). Studies using hepatoblastoma cells or human embryonic kidney (HEK) cells and HBx-expressing plasmid demonstrated p16^INK4a^ repression (Kim et al., 2010), p38^MAPK^ activation (Kim et al., 2007), or aberrant NFκB signaling (Shukla et al., 2011). These studies also showed cellular senescence induction by HBx (Idrissi et al., 2016). The elevation of IL-8 in HBV-infected patients PBMC or HBV-transfected cells (HepG2.2.15 cells) have been reported (Yu et al., 2011). Zhang et al. also reported the upregulation of IL-8 in HBV-associated HCC in both cell lines (HepG2, HepG2.2.15, or HepG2-hNTCP cells) and clinical specimens. This high IL-8 production was reportedly induced through mitogen-activated protein kinase kinase (MEK)-ERK signaling activation by HBx in HBx-overexpressing HEK cells (Zhang et al., 2021). In this regard, we performed capillary-based WB analyses of HBx protein; however, we failed to detect it even with a high sensitivity of capillary-based WB apparatus. On the other hand, we observed concentration-dependent chemiluminescence signals in the analysis of recombinant protein as well as in the experiments of p16^INK4a^ (Fig. 2B). Although we were unable to quantify HBx protein in HBV-infected human hepatocyte nuclei, HBx may account for in part of the reduced SA-β-Gal activity and dynamic changes in cell cycle regulatory proteins and transcriptional factors in human chronic HBV-infected hepatocytes treated with NAs. Moreover, HBx represses lysosomal maturation and suppresses hepatocellular autophagic degradation. Thus, HBx may contribute to the development of HBV-associated HCC (Liu et al., 2014).

Notably, we performed all studies using primary human hepatocytes and infectious live HBV, considered the best experimental systems reflecting physiological conditions. Our findings show that HBV infection induces ⅰ) decreased SA-β-Gal activity (which may reflect the repression of the lysosomal activity due to the chronic HBV infection), ⅱ) decreased cell cycle regulatory proteins p21^CIP1^ in nuclei (which may reflect the lower anti-carcinogenesis effect inducted by HBx), and ⅲ) elevated p38^MAPK^ in human hepatocytes *in vitro* and *in vivo*. These phenotypes are rescued by anti-HBV treatment, especially with *E*-CFCP. We hypothesize that the phenotypes are related to the activation or perturbation through p21^CIP1^ in the nucleus, which leads to HBV-infected cells back to the physiological phenotypes that are comparable to the HBV-uninfected cells. This hypothesis is supported by a recent study by Zai et al., who showed that early antiviral treatment could reduce the proteomic and transcriptomic cytopathic effect of long-term HBV infection in primary human hepatocytes (Zai et al., 2022). Although the exact mechanism by which NA therapy suppresses carcinogenesis and exerts its clinical effects remains unclear, the results of the studies in human hepatocytes suggest that a cell cycle regulatory protein such as p21^CIP1^ is implicated in the efficacy of the therapy. Moreover, as we have discussed above, the involvement of HBx is strongly presumed. In this regard, a notable limitation of the current study is the lack of HBx detection. Therefore, we could not directly prove our hypothesis. Due to its short half-life and potential unstructured region, understanding the role of HBx in the HBV life cycle remains a significant technical challenge. Thus, most studies have had to demonstrate the function of HBx in conditions of overexpression above biologically relevant levels (Slagle et al., 2015), and there are still difficulties in understanding the mechanisms of HBx during virus replication in systems reflecting physiological conditions (Slagle BL, Cold Spring Harb Perspect Med. 2016). Our *in vitro* and *in vivo* assay system using human hepatocyte and live HBV should provide insight to disrupt chronic HBV replication and to do so, further improvement of HBx protein detection should be needed to appraise detailed mechanisms of the phenotypes observed in the current study.

Taken together, the present data suggest that complete blocking of viral replication in host cells with highly potent and safe NAs may eventually prevent a subsequent course of pathological progression of HBV infection, including carcinogenesis.

## CRediT authorship contribution statement

**Yuki Takamatsu:** Conceptualization, Data curation, Formal analysis, Investigation, Methodology, Visualization, Writing – original draft, Writing – review & editing. **Sanae Hayashi:** Data curation, Investigation, Methodology, Writing – review & editing. **Hiroki Kumamoto:** Investigation, Resources, Writing – review & editing. **Shuhei Imoto:** Investigation, Resources, Writing – review & editing. **Yasuhito Tanaka:** Methodology, Supervision, Writing – review & editing. **Hiroaki Mitsuya:** Conceptualization, Funding acquisition, Methodology, Supervision, Writing – original draft, Writing – review & editing. **Nobuyo Higashi-Kuwata:** Conceptualization, Data curation, Funding acquisition, Investigation, Methodology, Project administration, Supervision, Writing – original draft, Writing – review & editing.

## Declaration of Competing Interests

The authors declare that they have no known competing financial interests or personal relationships that could have appeared to influence the work reported in this paper.

## Data Availability

Data will be made available on request.
